# No evidence of thyroid consequences in seven nuclear workers at the Tokyo Electric Power Company Fukushima Daiichi Nuclear Power Plant accident: 10-year follow-up results of thyroid status

**DOI:** 10.1093/jrr/rrac092

**Published:** 2023-01-04

**Authors:** Hideo Tatsuzaki, Riwa Kishimoto, Osamu Kurihara, Takako Tominaga, Shunichi Yamashita

**Affiliations:** National Institute of Radiological Sciences, Quantum Life and Medical Science Directorate, National Institutes for Quantum Science and Technology (QST), Chiba 263-8555, Japan; Diagnostic Radiology Section, Department of Diagnostic Radiology and Radiation Oncology, QST Hospital, Quantum Life and Medical Science Directorate, National Institutes for Quantum Science and Technology (QST), Chiba 263-8555, Japan; National Institute of Radiological Sciences, Quantum Life and Medical Science Directorate, National Institutes for Quantum Science and Technology (QST), Chiba 263-8555, Japan; National Institute of Radiological Sciences, Quantum Life and Medical Science Directorate, National Institutes for Quantum Science and Technology (QST), Chiba 263-8555, Japan; National Institute of Radiological Sciences, Quantum Life and Medical Science Directorate, National Institutes for Quantum Science and Technology (QST), Chiba 263-8555, Japan

**Keywords:** thyroid, Fukushima Daiichi Nuclear Power Plant (FDNPP) accident, follow-up, workers, cancer, thyroid function

## Abstract

Seven emergency nuclear workers, who had internal exposure due to an intake of radionuclides, mainly I-131, during the emergency response operation in March 2011, after the accident at the Tokyo Electric Power Company (TEPCO) Fukushima Daiichi Nuclear Power Plant (FDNPP), visited the National Institute of Radiological Sciences (NIRS) outpatient clinic for medical evaluation. They were followed up after their first visit for 10 years. The estimated committed equivalent doses to the thyroid were distributed between 3.2 to 1.2 × 10 Sv. This group thought to be received highest exposure at the accident. None of the workers had symptoms related to abnormal thyroid function. The examinations, including thyroid function tests and ultrasound, detected no abnormalities related to radiation exposure. However, there is a need for continuous monitoring of their thyroid status for longer periods in the future.

## INTRODUCTION

Late-onset thyroid consequences from radiation exposure, especially radioactive iodine intake during the emergency response, are a potential health problem after a nuclear accident. The risk of cancer induction is a major concern among the late effects. It is well known that the risk of thyroid cancer is elevated in those exposed during infancy and adolescence; however, there is controversy regarding whether older adults are vulnerable to internal exposure to thyroid glands. The absorbed dose to the thyroid is a primary determinant of the effect, regardless of external or internal exposure. According to an epidemiological study of Chernobyl liquidators, the excess relative risk per 100 mGy was 0.38, and a significantly increased risk was observed at doses of 300 mGy or above, suggesting the necessity of long-term follow-up for nuclear workers who received nearly or more than 300 mGy to their thyroid glands [1]. Furthermore, the effective and prompt implementation of iodine thyroid blocking (ITB) is important and should be considered before and during the release of radioactive iodine.

In Fukushima, Japan, following a large earthquake and tsunami on 11 March 2011, a serious combined disaster occurred at the Tokyo Electric Power Company (TEPCO) Fukushima Daiichi Nuclear Power Plant (FDNPP), releasing a large amount of radioactive materials, including mainly I-131, Cs-134 and Cs-137. The outline and details of the accident have been evaluated and reported by various international and Japanese organizations [2–7]. During the crisis phase of the accident, many emergency nuclear workers on site were required to engage in response operations to mitigate the accident and resolve the situation under serious environmental conditions. In addition, the Japan Ministry of Health, Labor and Welfare raised the occupational dose limit for these workers from 100 to 250 mSv in effective dose on 14 March 2011, which was terminated in mid-December 2011 [7]. This condition led some emergency nuclear workers to be exposed to radiation at relatively higher dose levels. According to the official report from TEPCO, 0.7% of the workforce (173 individuals) received a cumulative effective dose greater than 100 mSv [2]. Among them, seven were referred to our institute to evaluate the thyroid dose more precisely and follow up on their thyroid status for a long period. Therefore, in this report, we describe the 10-year follow-up changes in thyroid status to clarify any effects on the thyroid glands.

## MATERIALS AND METHODS

### Cases

After the early phase of the emergency response work at FDNPP, in March 2011, nuclear workers were examined by whole body counters in Onahama, Fukushima, operated by the Japan Atomic Energy Agency. Seven of these workers, who had high radiation activity in their bodies and suspected to have more than 250 mSv effective dose, were referred to the National Institute of Radiological Sciences (NIRS) in Chiba from Fukushima. All seven workers were on duty at the FDNPP on 11 March. The first emergency worker visited the NIRS outpatient clinic on 30 May 2011, and the last visited on 1 July 2011, 11–15 weeks after the estimated period of radionuclide exposure. They were examined at the NIRS and followed up afterward. The follow-up results until the end of January 2021 (almost 10 years) are reported in this article.

### Examinations

The examination included history taking, physical examination, blood tests (thyroid hormones: free T3 [FT3], free T4 [FT4] and thyroid stimulating hormone [TSH]), and neck ultrasound examination. We applied the diagnostic criteria used in the Fukushima ultrasound survey to report ultrasound results in this study [8]. The diagnostic criteria have four judgment categories: A1 category means no mass lesion, and A2 means solid nodule less than 5 mm or cystic lesion less than 20 mm.

### Dose assessments

*In vivo*, direct dose measurements for internal contamination with radionuclides were performed using a thyroid monitor with a highly purified Ge (HPGE) detector and a whole body counter equipped with six HPGE detectors in an iron shielding chamber. Before these, body surface contamination was examined using a Geiger Müller tube surface survey meter. The thyroid equivalent dose of internal exposure was calculated according to the International Commission on Radiological Protection (ICRP) bio-kinetic model [9] based on the measurement results at the first three visits. Thyroid doses were expressed by committed equivalent doses for 50 years calculated using the inhalation dose coefficients of ICRP, although the absorbed dose is suitable for considering tissue reactions. The absorbed and equivalent dose values were equal in these workers, as the radiation weighting factor for β and γ-rays from I-131 was 1. These doses were assessed based on reasonably conservative intake scenarios, in which radionuclide intake occurred on 11 March 2011, and the following 2–4 days. The equivalent doses to the thyroids of six of the seven workers have been reported [10]. (Note: ID alphabet symbols used for workers in the current report were not identical to this report.) The doses for an additional worker (worker F) were also calculated using the same methodological approach. Technical details of the measurements were reported in another article [11]. Effective dose from Cs-134, 136, 137 and Te-129 m, measured by the whole body counter, were between 0.3–10 mSv and almost two order smaller than those from I-131. Thus, contribution to thyroid dose from other radionuclides were not significant (partly reported in another article [11]). Regarding the external doses to the thyroid for the seven workers, the effective doses taken from the United Nations Scientific Committee on the Effects of Atomic Radiation (UNSCEAR) report [2] were referred to as an approximate value to the external thyroid equivalent dose in this report. Both internal and external doses were rounded to two significant figures.

### Follow-up

After the first several visits, the workers were followed up regularly at the NIRS for health checks (or at the National Institutes for Quantum Science and Technology [QST] as the successor organization). The health check frequency was once a year and increased depending on requests from the workers or decreased owing to their work schedules. The interval between health checks also decreased owing to travel restrictions by the COVID-19 pandemic after 2020. This study analyzed the data until June 2021, almost 10 years after the accident.

### Ethical consideration

Informed consent was obtained from the seven emergency nuclear workers according to the approval of the Institutional Research Ethics Committee (Approval no.: 12–010).

## RESULTS

The seven emergency nuclear workers were all men and worked in the FDNPP for mitigation and response operations from the first day of the accident. The age and first date of ITB are shown in Table 1. The date of the first ITB was based on the workers’ history.

**Table 1:** Emergency nuclear workers’ characteristics and radiation doses

Worker	Age at accident	sex	Date of first ITB	Committed equivalent dose to thyroid (Sv)	External exposure dose (mSv)	Total equivalent dose to the thyroid (Sv)
A	30s	M^a^	13 March	1.2 × 10	8.9 × 10	1.2 × 10
B	40s	M^a^	13 March	1.1 × 10	1.1 × 10*^2^*	1.1 × 10
C	20s	M^a^	11 March	7.5	4.4 × 10	7.5
D	50s	M^a^	14 March	4.6	1.1 × 10^2^	4.7
E	20s	M^a^	22 March	5.8	3.3 × 10	5.8
F	30s	M^a^	No administration	3.2	7.3 × 10	3.3
G	20s	M^a^	28 March	5.3	5.1 × 10	5.4

^a^M, male

None of the workers had any remarkable symptoms which were thought to be directly related to radiation exposure until the first visit to our hospital and thereafter. More than 1 month had passed from the start of the accident to the first visit. At each visit, measurements of the body surface contamination showed background levels; there were no detectable surface contaminations on all the workers at the first visit to our hospital and thereafter.

The radioisotopes detected using the whole body counter in these workers were I-131, Cs-134, Cs-136, Cs-137 and Te-129 m. Committed equivalent doses to the thyroid for 50 years based on NIRS dose assessment are indicated in Table 1. The estimated committed equivalent doses to thyroid were distributed between 3.2 to 1.2 × 10 Sv. The external doses and sum of internal and external doses are also indicated in Table 1. The total equivalent dose to the thyroid is distributed between 3.3 to 1.2 × 10 Sv. External exposure doses were two digits smaller than the internal exposure doses and contributed slightly to the total doses.

**Fig. 1:**
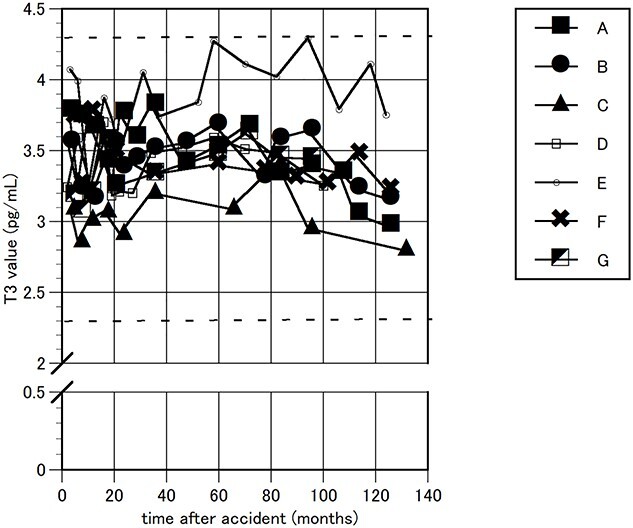
Changes in FT3. The time course of FT3 values is indicated for seven emergency nuclear workers. The time is indicated from the date of the earthquake. Two horizontal thick dotted lines indicate a normal range.

**Fig. 2:**
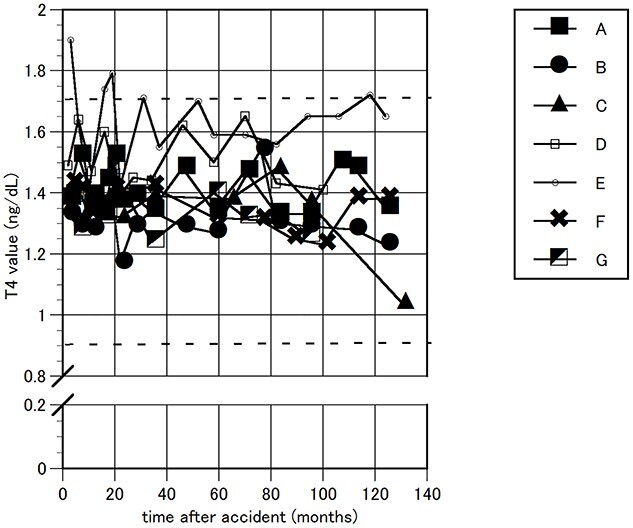
Changes in FT4. The time course of FT4 values is indicated for seven emergency nuclear workers. The time is indicated from the date of the earthquake. Two horizontal thick dotted lines indicate a normal range.

**Fig. 3:**
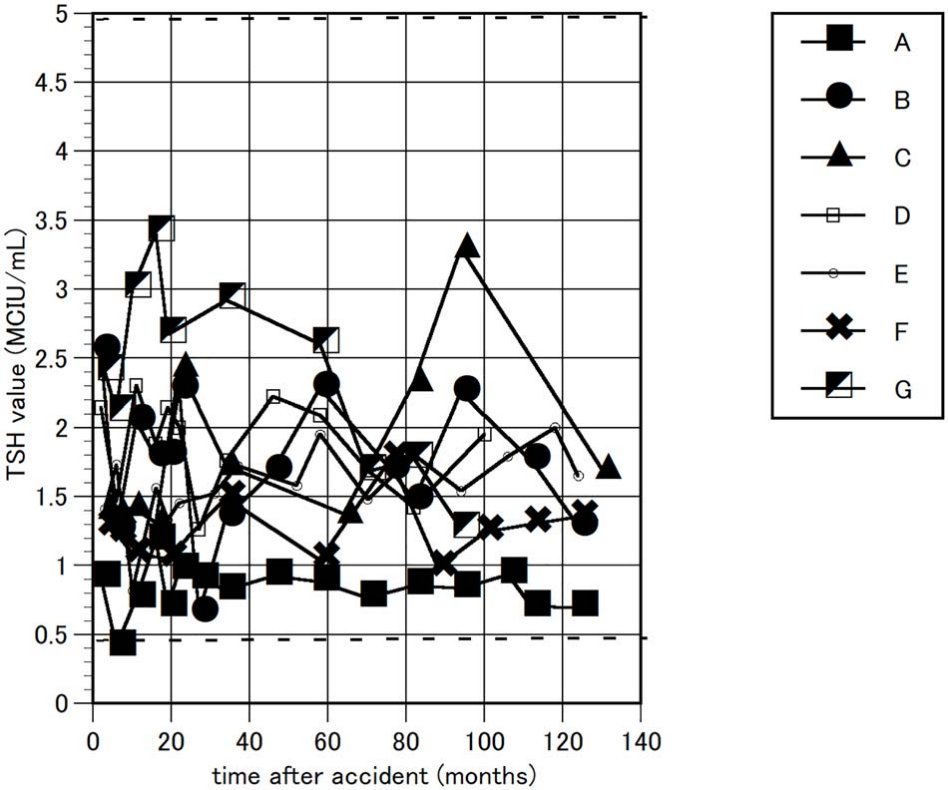
Changes in TSH. Changes in TSH values are indicated for seven emergency nuclear workers. The time is indicated from the date of the earthquake. Two horizontal thick dotted lines indicate a normal range.

The FT3, FT4 and TSH levels in the blood were measured as thyroid function tests. Their time courses are shown in Figs 1–3. During the follow-up period, FT4 showed a marginally high value in one worker, a maximum of 1.9 ng/dL (normal range 0.90–1.70 ng/dL); and TSH showed a marginally low value in another worker, 0.407 MCIU/mL, (0.500–5.00 MCIU/mL). These values were transient and returned to normal within a short period. Otherwise, all FT3, FT4 and TSH levels were maintained within the normal range.

Thyroid ultrasound examination was performed at least once in 2 years during the follow-up period, except after 2020, when movement in Japan was restricted owing to the COVID-19 pandemic. Because the examination results, such as the mass lesion size, changed slightly during the time course of the follow-up possibly due to measurement errors, the most remarkable abnormalities during the follow-up period are reported in Table 2. Among the seven workers, no mass lesions were detected in one, small cysts less than 20 mm in diameter were detected in five, and multiple small cysts and nodules compatible with adenomatous goiter were found in one. The last one is not related to radiation exposure from the accident because the finding was detected during the first examination and did not change in the following period. Using the diagnostic criteria of thyroid nodules and cysts from the Fukushima Health Management Survey [8], the highest criteria during the follow-up course are shown in Table 2. One worker met criteria A1, and six met A2 (Table 2). The thyroid is enlarged when the largest diameter exceeds 60 mm. The size increased slightly for three workers. In one, it was observed at the first visit and continued to be stable afterwards. In the other two, enlargement was in the marginal range with no tendency for continuous enlargement. Thus, there were no abnormal findings or changes thought to be related to radiation exposure.

**Table 2:** Ultrasound findings in worst examination during the follow-up period

Worker	Findings in internal structures / size of the gland^a^	Criteria
A	Small cystic lesions up to 7.5 mm/slightly enlarged.	A2
B	No mass lesion/no enlargement.	A1
C	Hypoechoic mass of 2 mm suspected/slightly enlarged.	A2
D	Small cystic lesions up to 3.7 mm/no enlargement.	A2
E	Small cystic lesions up to 3 mm/no enlargement.	A2
F	Multiple small nodules compatible with adenomatous goiter, small cystic lesion up to 5 mm/slightly enlarged.	A2
G	Small cystic lesion up to 6 mm/no enlargement.	A2

^a^The largest lobe length of more than 60 mm is considered enlargement.

## DISCUSSION

The reflections and lessons learned from the Chernobyl Nuclear Power Plant accident demonstrate that internal exposure, besides external exposure to thyroid glands, is a major risk factor for late-onset thyroid cancers in addition to other confounding factors [12, 13]. Since the dose exposed to the thyroid gland is critical to evaluate the relationship between radiation and thyroid consequences, we should at first discuss the route and causes of internal exposure for seven emergency nuclear workers. Discussing the intake cause is also important for preventing internal exposure in any future accident. Radionuclide intake can occur via three possible routes: ingestion, inhalation or through injured skin. Inhalation is considered the main route of radionuclide intake in the described workers on site. Emergency nuclear workers wear protective suits and equipment in principle, including respiratory protection. The workers in this study wore full-face masks when they worked in and around the reactor buildings; however, they could not completely avoid internal contamination and indeed received high internal doses to the thyroid glands (range; 3.2–12 Sv, *n* = 7). These doses though to be the highest among all workers. Thus, these workers have the highest possibility to develop radiation injuries among all workers. Although such causes of intake or occasions of intake were not identified clearly, there are four possible reasons of inhalation in considering the history and behavior of each worker. First, the inhalation at the time of detaching the full-face masks in the main control rooms of the FDNPP where they rested. They detached masks when they ate and drank; however, air with a high radioactive iodine concentration entered from the outside owing to the damage to the airtight door by the reactor unit 1 explosion. Second, a leak through a mask-skin gap, especially around the sidepieces of the glasses, during their emergency response. Contamination of the skin surface near the sidepieces was detected in two workers when they went out from the controlled area after working with a full-face mask. Third, the use of inappropriate filters for the masks. Although charcoal filters should have been used to remove iodine, normal dust filters were used for these workers in the first phase before the arrival of proper filters. Fourth, ingestion when they ate and drank in the main control room with possible contamination from the air with radionuclides, as described above. Their recall of memories of the action during the response operation were, however, inaccurate, and dose calculation could have uncertainties. Thus, recall bias should be considered. A single cause could not be identified; however, these cases taught us how to protect and prevent unpredicted exposure from inhalation after nuclear accidents. These cases suggest that the preparation of proper protective wear and equipment and their proper usage should be strictly taught to all potential emergency nuclear workers in nuclear power plants ahead of accidents during normal operational periods.

Another important point is the type of exposure scenario, which is appropriate and applicable for accurate dose estimation. The time course of intake, or intake scenario, is a major factor influencing dose calculation. The dose assessment of six out of seven workers was reported in a previous study [10]. The dose assessment of another worker followed the same methodological approach. This study used a conservative approach, assuming intake between 11–12, 11–13 or 11–14 March. The major release occurred after 12 March, when the first hydrogen explosion of Unit 1 occurred, until the end of March [2]; therefore, these scenarios could cause overestimation.

According to the UNSCEAR report, radiologically significant radionuclides released into the atmosphere are Te-132, I-131, Te-132, I-133, Xe-133, Cs-134, Cs-136 and Cs-137. [2]. Noble gases such as Xe-133; however, do not accumulate in the body and internal exposure to it is not large, and Cs does not largely contribute to the thyroid dose. Short-lived radioiodines, Te-132/I-132 and I-133, could contribute to the thyroid dose; however, these data are lacking. Although the primary radionuclide contributing to the workers’ thyroid exposure was I-131, the doses indicated in this report may have been underestimated [2, 14].

The possible health effects of the I-131 dose accumulated in thyroid glands include hypothyroidism, thyroiditis and autoimmune disease known as deterministic effects or tissue reactions. In addition, thyroid cancer can be induced, which is a well-known stochastic effect. Benign thyroid nodules have also been reported [15]. The incidence of childhood thyroid cancer increases a few years after exposure [16]. Therefore, a large-scale thyroid screening with ultrasound examination for neonates, infants and young adolescent residents at the time of the accident was conducted by Fukushima prefecture in the framework of the Fukushima Health Management Program [17]; however, a direct comparison of these residential data is inappropriate because the age distribution and dose range were quite different from the workers in this study. The expert group for International Agency for Research on Cancer recommends a long-term thyroid monitoring program for higher risk individuals after a nuclear accident [18]. A longer follow-up is necessary for these workers to detect possible thyroid cancers.

Some studies have reported a threshold for radiation-induced hypothyroidism. Hypothyroidism is caused by radiation exposure of more than several Gy [19]. Direct damage to the thyroid gland following neck radiation at a fractionated dose of >18 Gy commonly presents as hypothyroidism, with low thyroxine and elevated TSH levels [15]. Conard *et al.* reported hypothyroid cases with exposure at 1 year of age at Marshal Island nuclear explosion with an estimated dose of 7–14 Gy to the thyroid [20]. In a review of previous studies, Reiners *et al.* concluded that the dose threshold for hypothyroidism after external beam radiotherapy is 10 Gy, while I-131 internal radiotherapy for Graves’ disease rarely causes hypothyroidism below 50 Gy [21]. Criteria for mitigation interventions have also been proposed. To avoid or minimize severe deterministic effects, the International Atomic Energy Agency indicated exposure doses as ‘generic criteria’ and recommended taking protective action if internal exposure from acute radionuclide intake exceeds 2 Gy in 30 days [22]. This recommendation can be interpreted as any symptom unlikely to occur with exposure to less than 2 Gy; however, the target population includes all ages. Although the workers in this study received a thyroid dose over some of these threshold levels, the dose was below the level for internal radiotherapy in adults. In patients treated with internal radiotherapy, proportion of hypothyroidism increased from 10 to 25 years [23]. Although no hypothyroidism or thyroid malfunction due to radiation was observed during the 10-year follow-up period, a longer follow-up is necessary.

In addition to reducing the amount of radioactive iodine intake, ITB is a well-known prophylactic countermeasure for reducing thyroid injury risk [24]. A sufficient amount of stable iodine is recommended for ITB in the event with the release of radioactive iodine [25]. Oral administration of Potassium iodide (KI) is commonly used in most countries. ITB is effective if administered up to 2 days before the intake. This means the effective period is limited [26, 27]. If the time relationship between the medication and inhalation is within the proper range, prophylaxis could have some effects. Most workers in the current study took stable iodine pills at some stage. However, they did not use ITB at an earlier time, partly because the drugs were not available in the control room where they worked in the early phase. The timing of intake is not clear, and the analysis of possible effects of ITB is difficult. However, it is likely that a part of the intake occurred before the start of ITB, as the atmospheric release of radionuclides started on the morning of 12 March. Despite some protective measures, the thyroid doses of workers in this study were relatively high. In contrast, ITB has some side effects, as reviewed by Spallek *et al.* [28]. In a large-scale administration to residents in Poland, medically significant adverse reactions were reported in 0.2% of residents taking the medication [29]. In the current study, the workers did not experience any symptoms when taking iodine pills.

## CONCLUSION

Among seven emergency nuclear workers with radionuclide intake during mitigation work at the FDNPP accident during a follow-up of nearly 10 years, no worker showed thyroid consequences due to internal radiation exposure. These workers thought to receive the highest dose among all workers. This result agrees with the data obtained after internal radiotherapy. Longer observation periods are required, especially for evaluating any possibility of late-onset thyroid cancer and hypothyroidism. In addition, a single intake route for their thyroid exposure was not identified; however, appropriately preventive use of ITB and proper protective equipment could have reduced internal exposure to the thyroid.

## Data Availability

The original data are in the medical record of the institute; thus, these data are not available to outside.
